# Degradation of Kidney and Psoas Muscle Proteins as Indicators of Post-Mortem Interval in a Rat Model, with Use of Lateral Flow Technology

**DOI:** 10.1371/journal.pone.0160557

**Published:** 2016-08-23

**Authors:** Dong-Gi Lee, Kyeong Eun Yang, Jeong Won Hwang, Hwan-Soo Kang, Seung-Yeul Lee, Seoyeon Choi, Joonchul Shin, Ik-Soon Jang, Hyun Joo An, Heesun Chung, Hyo-Il Jung, Jong-Soon Choi

**Affiliations:** 1 Biological Disaster Analysis Group, Korea Basic Science Institute, Daejeon, Republic of Korea; 2 National Core Research Center for Nanomedical Technology, Yonsei University, Seoul, Republic of Korea; 3 Graduate School of Analytical Science and Technology, Chungnam National University, Daejeon, Republic of Korea; 4 School of Mechanical Engineering, Yonsei University, Seoul, Republic of Korea; University of Glasgow, UNITED KINGDOM

## Abstract

We investigated potential protein markers of post-mortem interval (PMI) using rat kidney and psoas muscle. Tissue samples were taken at 12 h intervals for up to 96 h after death by suffocation. Expression levels of eight soluble proteins were analyzed by Western blotting. Degradation patterns of selected proteins were clearly divided into three groups: short-term, mid-term, and long-term PMI markers based on the half maximum intensity of intact protein expression. In kidney, glycogen synthase (GS) and glycogen synthase kinase-3β were degraded completely within 48 h making them short-term PMI markers. AMP-activated protein kinase α, caspase 3 and GS were short-term PMI markers in psoas muscle. Glyceraldehyde 3-phosphate dehydrogenase (GAPDH) was a mid-term PMI marker in both tissues. Expression levels of the typical long-term PMI markers, p53 and β-catenin, were constant for at least 96 h post-mortem in both tissues. The degradation patterns of GS and caspase-3 were verified by immunohistochemistry in both tissues. GAPDH was chosen as a test PMI protein to perform a lateral flow assay (LFA). The presence of recombinant GAPDH was clearly detected in LFA and quantified in a concentration-dependent manner. These results suggest that LFA might be used to estimate PMI at a crime scene.

## Introduction

Estimation of post-mortem interval (PMI) is considerably important practical task in forensic science. Estimating the exact time of death by classical way is limited by simple observations such as *livor mortis*, *algor mortis*, and *rigor mortis*. An exact estimate of PMI is a very critical and important task in forensic investigations. Although technologies to determine the PMI have been developed for more than a century, still it remains difficult to satisfy all the conditions. The time of death was generally defined by measuring body temperature post-mortem using possible algorithms to obtain a time equation for body cooling behavior. However, numerous variables affecting PMI must be considered to approximate time of death [1]. Several analytical methods such as magnetic resonance spectroscopy, flow cytometry, and immunohistochemistry have been developed to assess PMI [2]. However, the accuracy of the estimate of PMI mainly depends on external variables such as environmental temperature, air flow, body structure, location, and cause of death.

Biochemical assessments of PMI using electrolyte concentrations, enzyme activities, forensic entomological evidence, and RNA degradation profiling have been used in criminal investigations [3–6]. In particular, the analysis of time-dependent nucleic acids (DNA and RNA) has been widely used in the forensic field [7]. RNA degradation profiling of body fluid is currently a focus of the forensic scientific community as a way to determine the PMI [8–10]. In general, RNAs are rapidly degraded by internal or external ribonucleases, so the PMI can be estimated within 1 day. As *in vivo* integrity and stability of RNAs differ among tissues and organs after death, post-mortem physiobiochemistry such as molecular biology and immunohistochemistry should be considered for practical use.

Degradation of proteins after death is slower and more reproducible than degradation of RNAs. In addition, mRNA expression is not correlated with protein expression in large parts of organs or tissues because of the versatility of post-transcriptional and post-translational modifications [11–13]. Post-mortem protein degradation has been studied in rat and human brains and showed that myelin basic protein, adenylate cyclase, and carboxymethylase are stable within 24 h post-mortem, whereas certain types of G-proteins and neurofilament proteins decrease as PMI proceeds [14–18].

In this study, we analyzed the post-mortem degradation of proteins from rat kidney and psoas muscle. The reason why these tissues were chosen is that it is located deeply inside the body; thus, it is less affected by a change in external temperature. Some signaling protein components, glycogen-regulating enzymes, and housekeeping protein were selected as PMI protein candidates according to the previous studies of mRNA degradation profiles [6, 19]. Furthermore, a lateral flow assay (LFA)-based diagnostic chip was used to possibly determine the PMI using the recombinant rat GAPDH protein as a pilot attempt. Lateral flow technology for estimating PMI might be developed to be useful at the crime scene for forensic application.

## Materials and Methods

### Experimental animals

Sprague–Dawley rats (male, 8 weeks, body weight; 250–300 g) were obtained from Daehan Bio-Link Inc. (Eumsung, Republic of Korea). Rate were maintained in the experimental animal facility at Korea Basic Science Institute. Rats were fed a normal diet and water with *ab libitum* for a week. And then, rats were immediately euthanized with carbon dioxide gas. The carcasses were assigned to one of eight PMI groups (n = 6 and n = 5 for rat kidney (total 48 rats) and psoas muscle (total 40 rats) per group, respectively). The sacrificed whole bodies were kept in a temperature-controlled room at 23 ± 1°C for 0, 24, 36, 48, 60, 72, 84, and 96 h. At the designated time of PMI, the kidneys and psoas muscles were removed and quickly frozen in liquid nitrogen. The organs were stored at −80°C prior to biochemical analyses. The animal care and experimental procedures used in this study were approved by the Animal Care Committee of Korea Basic Science Institute and followed the protocol of the Korean Council on Animal Care (KBSI-AEC 1306).

### Western blot analysis

Frozen whole kidneys or psoas muscles were crushed in a mortar in liquid nitrogen. The powder was suspended in PRO-PREP Protein Extraction Solution (iNtRON Biotechnology, Inc., Republic of Korea) and incubated for 30 min on ice. The samples were centrifuged at 13,000 rpm for 30 min, and the supernatant was collected. Protein concentration was quantified using the Bradford Assay Kit (Bio-Rad, Hercules, CA) employing bovine serum albumin as the standard according to the manufacturer’s instructions. Total protein of 30 μg from each sample was mixed with protein loading buffer and denatured using heating block at 95°C for 5 min with tightly sealed lid. And then, the denatured proteins were resolved on sodium dodecyl sulfate-polyacrylamide gel electrophoresis and transferred to a nitrocellulose membrane (Pall Corp, Cortland, NY). The blots were blocked with 5% (w/v) skimmed milk powder in Tris-based saline buffer containing 0.05% (v/v) Tween-20 (TBST) for 1 h, probed with antibodies against GAPDH (1:6000 dilution, sc-25778, Santa Cruz Biotechnology, Santa Cruz, CA), caspase-3 (1:1000 dilution, #9662, Cell Signaling Technology, Beverly, MA), peroxisome proliferator-activated receptor gamma (PPAR-γ) (1:1000 dilution, PAB14848, Abnova Co., Taipei, Taiwan), glycogen synthase (1:4000 dilution, #3886, Cell Signaling Technology), glycogen synthase kinase-3β (GSK-3β) (1:1000 dilution, sc-8257, Santa Cruz Biotechnology), p53 (1:2000 dilution, sc-126, Santa Cruz Biotechnology), AMP-activated protein kinase α (AMPKα) (1:2000 dilution, #2532, Cell Signaling Technology), and β-catenin (1:1000, sc-7963, Santa Cruz Biotechnology) at 4°C overnight. Subsequently the blots were incubated with secondary antibody horseradish peroxidase (HRP)-conjugated anti-rabbit IgG (sc-2004, Santa Cruz Biotechnology) or anti-mouse IgG (sc-2005, Santa Cruz Biotechnology) for 2 h at room temperature. Signals were detected with the Immobilon Western Chemiluminescent HRP substrate (Millipore, Bedford, MA) and the ImageQuant LAS 4000 mini (GE Healthcare, Little Chalfont, UK). Signal densities were quantified by ImageJ software (http://rsbweb.nih.gov/ij). Relative quantification was calculated based on the signal intensity of the 0 h PMI immunoblot as a control.

### Immunohistochemistry

Immunohistochemical staining was performed according to the protocol of Symonowicz et al [20]. Rat kidney and psoas muscle tissues were fixed in 4% (v/v) formaldehyde in PBS for 24 h. The tissues were embedded in paraffin and cut into 4 μm wide slices. After deparaffinization and rehydration, endogenous peroxidase activity was blocked with 3% (v/v) hydrogen peroxide. Antigen was retrieved by pressure cooking the sections for 10 min in citrate buffer (10 mM citric acid, 0.05% Tween 20, pH 6.0) in a microwave. The sections were blocked for 1 h at room temperature with 5% (w/v) bovine serum albumin (BSA) to avoid nonspecific staining and incubated with primary anti-caspase-3 (1:200 dilution, #9662, Cell Signaling Technology) and anti-glycogen synthase (1:1000 dilution, #3886, Cell Signaling Technology). The antigen-antibody complex was detected by the labeled streptavidin-biotin-peroxidase method using a Histostain-plus 3^rd^ Gen IHC detection kit (Zymed, San Francisco, CA). Immunohistochemically stained sections were counterstained with hematoxylin (Merck, Darmstadt, Germany). Slides were scanned using Imager Z2 (Carl Zeiss, Zena, Germany) with TissueFaxs ver. 3.0 software (TissueGnostics, Vienna, Austria).

### Cloning and recombinant protein expression of GAPDH

Total RNA was isolated from kidney tissue using TRI reagent (Sigma-Aldrich, St. Louis, MO) and used for the first cDNA synthesis. The 1002 bp DNA fragment was amplified by polymerase chain reaction (PCR) using a pair of rat GAPDH specific primers (forward, 5’-CGCGCGGCAGCCATATGGTGAAGGTCGGTGTAAAC-3’; reverse 5’-GGTGGTGGTGCTCGAGTTATTCTTTGCTCGCCATG-3’) extended at 5’ to incorporate restriction sites shown in underlines for *NdeI* and *XhoI*, and sequenced for confirmation. The PCR product was cloned to pET28a protein expression vector (Novagen, Darmstadt, Germany). Competent cells of *E*. *coli* BL21 (DE3) (Novagen) were transformed with rat GAPDH-pET28a recombinant vector. Overexpression of rat GAPDH was achieved by induction with 0.2 mM IPTG for 16 h at 18°C. The recombinant GAPDH (rGAPDH) protein was purified to homogeneity by repeated Ni-NTA affinity chromatography and dialyzed with storage buffer (50 mM Tris-HCl, pH 8.0, 200 mM KCl).

### Sandwich enzyme-linked immunosorbent assay (ELISA)

A sandwich ELISA to detect the rGAPDH as a mid-term PMI marker in rat kidney was performed using different epitope polyclonal antibodies. Briefly, the ELISA plates were coated with 50 μL goat polyclonal GAPDH antibody (0.5 μg/mL, sc-20357, Santa Cruz Biotechnology) and incubated at 4°C overnight. Tris-base saline containing 1% (w/v) BSA was added to prevent non-specific binding for 2 h at room temperature. The plates were washed with TBST buffer. Serially diluted rGAPDH proteins are added to the plates at 0–100 ng/μL and incubated for 1 h 30 min at 37°C. After washing, rabbit polyclonal GAPDH antibody (1:1000, sc-25778, Santa Cruz Biotechnology) was added and incubated for 2 h at room temperature followed by washing. Bound antibodies were detected with HRP-conjugated goat anti-rabbit IgG antibody (1:1000, sc-2004, Santa Cruz Biotechnology) and revealed using 3,3’,5,5’-tetramethylbenzidine dye. Absorbance was measured at 450 nm using a microplate reader (Sunrise^TM^, Tecan, Basel, Switzerland).

### Lateral flow assay (LFA) of rGAPDH

The rGAPDH was assayed using a lateral flow immunosensor system previously used for alpha-amylase [21]. Briefly, 1% (w/v) BSA was added to the sample pad and dried for 60 min at 37°C. Goat-polyclonal GAPDH antibody (1 μg) conjugated with 30 nm gold nanoparticles were dispensed onto the conjugation pad. The pad was fully dried for 1 h at 37°C. Rabbit-polyclonal GAPDH antibody and goat anti-rabbit IgG antibody were dispensed into the test and control lines, respectively. After drying, the pads were assembled on the PVC plastic support pad in the following order; nitrocellulose membrane, conjugation pad, sample pad, and absorption pad. For the optimization and quantitation experiments, the procedure of the rGAPDH test is the following: first, the various concentrations of rGAPDH solution were applied to the strip. Then, the red-green-blue composition image of the assay results was recorded. The peak area of the red bands was read using the ImageJ software and the detection limit for rGAPDH was calculated as three standard deviations of the peak area.

### Statistical analysis

GraphPad Prism software (GraphPad, San Diego, CA) was used for the statistical analysis. All data were processed to assess differences between each PMI time-point and control PMI 0 using Student’s *t*-test. The values of PMI_50_ were determined as the time-point corresponding to the 50% expression level compared to that of control PMI-0 h according to the initial protein degradation linear regression equation. Statistical *P*-value < 0.05 was considered significant.

## Results

### Western blot analysis of the PMI proteins

We chose eight protein candidates related to glycogen-regulating enzymes, signaling protein components, and a housekeeping gene to profile post-mortem protein degradation [22–24].

**Fig 1:**
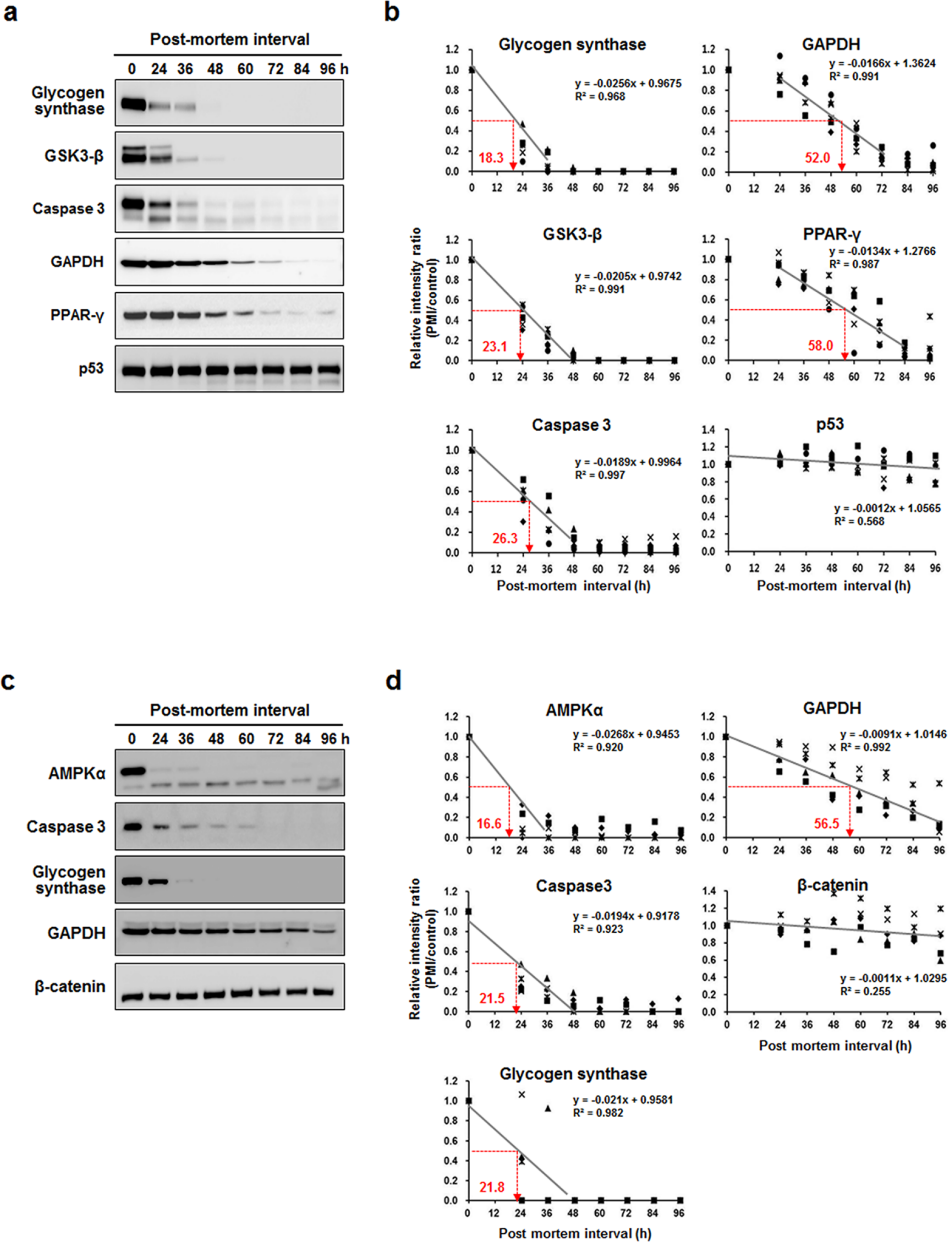
Degradation profiles of selected rat post-mortem interval (PMI) proteins. (a) Immunoblot analysis of kidney PMI proteins up to 96 h post-mortem. (b) Linear regression fits for kidney PMI protein degradation. PMI_50_ values (number beside the dotted arrow), indicating the 50% degradation time-point of the starting expression level, were determined by the linear regression curve. (c) Immunoblot analysis of psoas muscle PMI proteins up to 96 h post-mortem. (d) Linear regression fits for psoas muscle PMI protein degradation. Each data point represents five biological replicates.

The rat kidney or psoas muscle proteins extracted 0–96 h post-mortem at 12 h intervals were used for the quantitative comparison to observe post-mortem protein degradation profiles by Western blot analysis. The expression levels showed the different decay patterns of the specific proteins (Fig 1, S1 and S2 Figs). The protein degradation patterns were divided clearly into three groups: short-term, mid-term, and long-term PMI. Most of the selected PMI proteins regressed linearly with high R^2^ values, except p53 and β-catenin. The half maximum intensity of intact protein is referred to as the PMI_50_ based on a linear regression. A short-term PMI marker was defined as a protein showing a PMI_50_ value within 24 h post-mortem. The short-term PMI proteins from kidney included GS (PMI_50_ value = 18.3 h) and GSK3-β (PMI_50_ value = 23.1 h), as shown in Fig 1B. These short-term PMI proteins were completely degraded by 48 h post-mortem. In psoas muscle, GS (PMI_50_ value = 21.8 h) as well as AMPKα (PMI_50_ value = 16.6 h) and caspase 3 (PMI_50_ value = 21.5 h) was also classified as short-term PMI protein (Fig 1D). A mid-term PMI marker was defined as a protein with a PMI_50_ value between 24 h and 96 h post-mortem. The mid-term PMI proteins for kidney included caspase-3 (PMI_50_ value = 26.3 h), GAPDH (PMI_50_ value = 52.0 h), and PPAR-γ (PMI_50_ value = 58.0 h). The mid-term PMI proteins were completely degraded 84–96 h post-mortem. GAPDH (PMI_50_ value = 56.5 h) was also classified as mid-term PMI protein in psoas muscle. However, the degradation patterns of p53 and β-catenin seemed to be later than 96 h post-mortem, suggesting that these proteins were long-term PMI proteins (Fig 1B and 1D).

### Verification of protein degradation by immunohistochemistry

**Fig 2:**
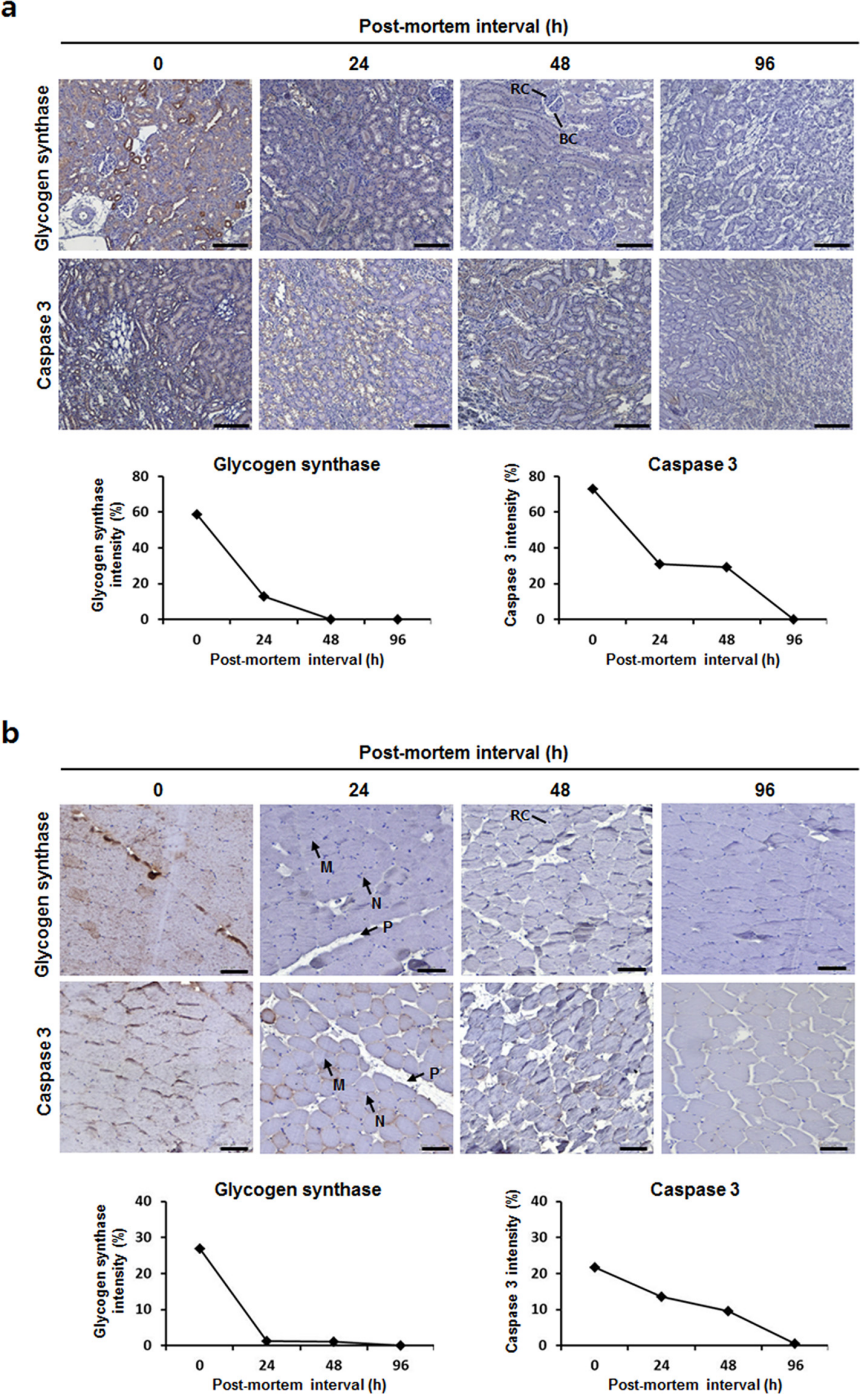
Immunohistochemistry and degradation curves of post-mortem rat tissues. Microscopic photographs of rat (a) kidney and (b) psoas muscle at the indicated PMI time-point. Degradation curves for glycogen synthase and caspase-3 expressions were obtained from TissueFACS scattergrams. RC, renal corpus; BC, Bowman’s capsule; M, muscle fiber bundle; N, nuclear; P, perimysium; Scale bar = 100 μm.

We observed protein degradation during the PMI by immunohistochemistry (IHC) to verify the biochemical PMIs of some proteins degraded in rat kidney or psoas muscle. Horizontal or vertical thin sections of rat kidney were stained with 3,3’-diaminobenzidine (DAB)-conjugated IHC-antibody GS or caspase-3, respectively. As shown in Fig 2A, the tightly organized kidney seemed to be slightly loosely organized as time proceeded after death. In the control (PMI 0 h) rat kidney, 60% of the space in the designated area was stained with DAB of which represented GS. However, only 10% of the area was stained with DAB in 24 h post-mortem, and none was stained for GS in 48 h post-mortem. A total of 76% of the DAB-stained area for caspase-3 was gradually decreased 48 h post-mortem and completely disappeared at 96 h post-mortem (Fig 2A, lower panel).

Horizontal thin section of rat psoas muscle was stained with GS or caspase 3 antibodies. The well-organized muscle tissues seemed to be slightly shrinking that it is more observed perimysium as time processed after death (Fig 2B). The DAB-stained area for GS in control psoas muscle was almost disappeared after 24 h post-mortem (Fig 2B, lower panel). In meantime, caspase-3 stain area was gradually decreased up to 96 h post-mortem. The decay patterns of GS and caspase-3 between Western blotting and IHC analyses were similar. These results indicate that the results of GS and caspase-3 IHC coincided with Western blot results from the indicated PMI time-point. Thus, a biochemical determination of PMI can be applied to forensics.

### Recombinant expression and purification of rat GAPDH

**Fig 3:**
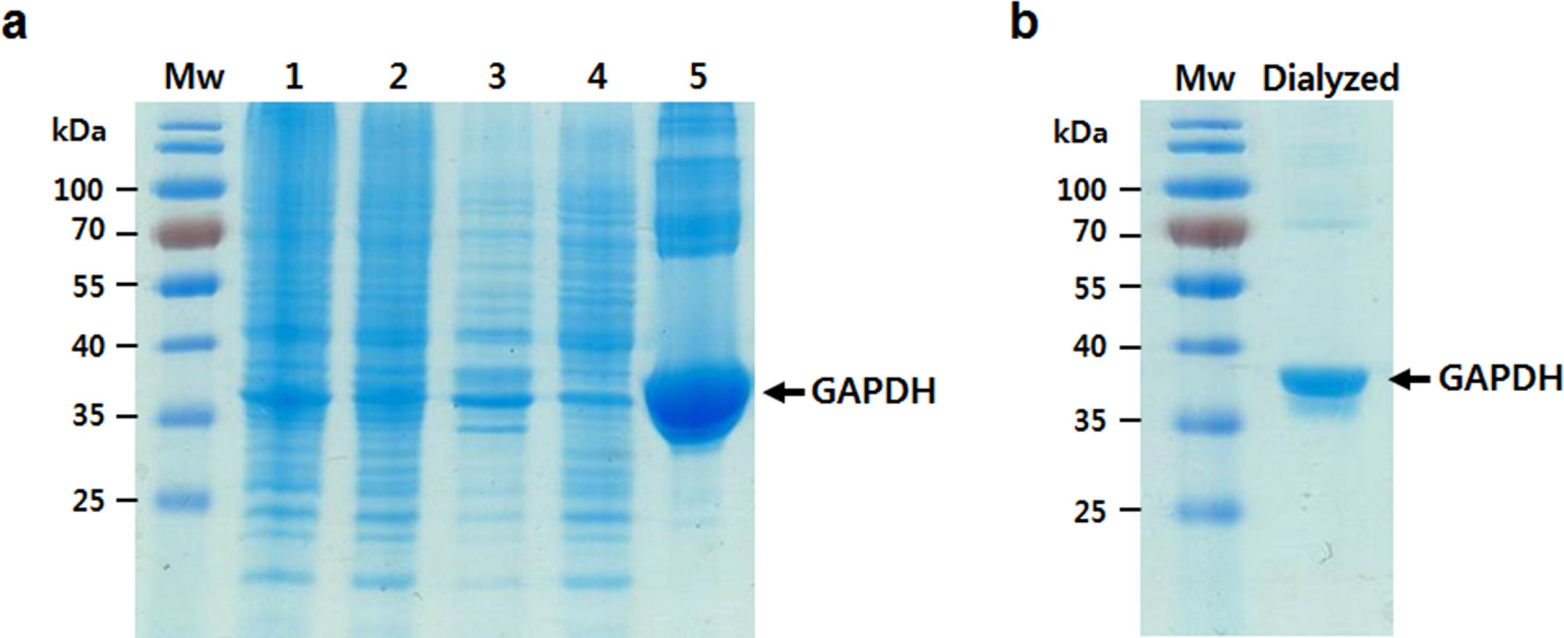
SDS-PAGE analysis of recombinant GAPDH expression. Coomassie Brilliant Blue-stained proteins were shown from (a) the serial purification procedure and (b) the final dialysis of rGAPDH. Under optimized condition, *E*. *coli* strain BL21 (DE3) was transformed with rat GAPDH-pET28a, and following IPTG induction resulted in the expression of GAPDH (37 kDa). Mw, protein molecular weight; 1, total lysate of transformed *E*. *coli*; 2, soluble supernatant fraction of total lysate; 3, insoluble pellet fraction of total lysate; 4, flow-through after affinity chromatography; 5, purified rGAPDH.

The full length GAPDH of rat kidney was transformed into *E*. *coli* BL21 (DE3) cell by pET28a vector system and overexpressed as a 6x-His-tag fusion protein. SDS-PAGE was employed to detect a target protein (Fig 3A). The recombinant GAPDH protein was purified using Ni-NTA affinity chromatography. In addition, purified recombinant protein was dialyzed with storage buffer (Fig 3B), and the concentration of the soluble recombinant protein was approximately 5.9 mg/mL. The purified rGAPDH protein was used for sandwich ELISA and LFA analyses to reveal the concentration-dependent antigen-antibody reaction properties.

### Detection of rGAPDH using the lateral flow immunosensor

**Fig 4:**
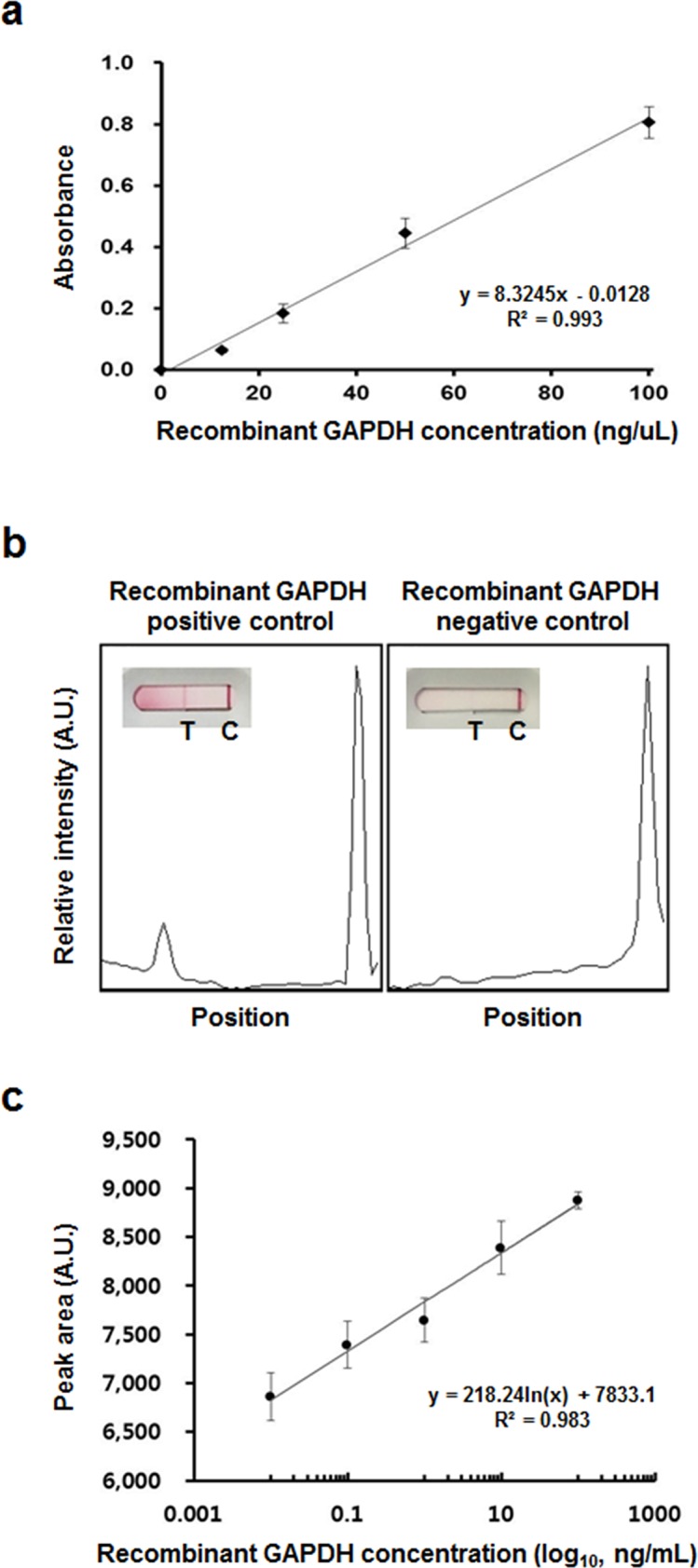
Lateral flow assay platform for PMI-indicating protein. (a) Standard calibration of rGAPDH concentration by sandwich ELISA assay. (b) Typical rGAPDH strip images and the corresponding signals in the presence of 0 and 100 ng/mL rGAPDH. The peak areas of red lines on the control (C) and the test line (T) were calculated using the ImageJ software. (c) Calibration curve of different concentrations of rGAPDH. The peak areas of red bands on the test line were calculated using the ImageJ software. Error bars are the standard deviation of triplicate experiments. A.U., arbitrary unit.

A sandwich ELISA assay was used with the GAPDH mid-term PMI marker of kidney to apply the PMI protein markers to LFA. The ELISA was used to measure test antigen concentrations in a sample. A sandwich ELISA is very rapid and efficient to detect whether an antigen is present in a sample. Relative concentration-to-intensity values showed the good correlations based on a serial dilution of the rGAPDH protein (R^2^ = 0.993) (Fig 4A). In the presence of rGAPDH, the red bands clearly appeared on the control and test line in an rGAPDH lateral flow immunosensor as expected. However, no red band, in the absence of recombinant GAPDH, was observed as shown in Fig 4B. The difference of the peak area was presented by positive (Fig 4B, left panel) and negative control (Fig 4B, right panel). The detection time was enough within 15 min. For the practicability of the rGAPDH lateral flow immunosensor, the calibration curves for the dependence peak area were obtained from rGAPDH concentration (in the range of 0.01–100 ng/mL). The detection of rGAPDH was applied at control and test line, respectively. The quantification was performed by calculating the peak area of red bands using ImageJ software. The peak area of the resultant red color band should vary proportionally to the concentration of rGAPDH. The linear regression equation for rGAPDH was Y = 218.24ln(x) + 7833.1 (R^2^ = 0.983) over the concentration ranges of 0.01–100 ng/mL and the detection limit of rGAPDH was revealed as 0.01 ng/mL (Fig 4C). As a result, the sensitivity and linear ranges of the rGAPDH lateral flow immunosensor are adequate and applicable for measuring post-mortem intervals.

## Discussion

The previous studies described reliable semi-quantitative protein methods to investigate PMI using post-mortem human tissue samples [25–28]. Identification of human PMI protein markers can be applied to indicate time after death with simplified human PMI determination kit based on lateral flow assay. Thus, the determination of PMI markers is required of considering various conditions including diverse post-mortem tissues and environmental temperature. We have initially investigated to identify various potential PMI protein markers in rat kidney and psoas muscle with controlled temperature up to 96 h post-mortem. We examined the protein degradation profiles of selected proteins in the rat kidney and psoas muscle to determine whether PMI proteins approximately indicate time after death. We chose soluble enzymes and typical signaling proteins such as GS, GSK-3β, caspase-3, GAPDH, PPAR-γ, AMPKα, p53, and β-catenin.

GS is a key enzyme in glycogen synthesis and metabolism as it converts glucose to glycogen, primarily in the liver and muscle. GSK-3 is a highly conserved serine/threonine protein kinase comprised of two isoforms such as GSK-3α and GSK-3β, which are glycogen synthase regulators and involved in several neurodegenerative diseases [29, 30]. AMPK is well-known a serine/threonine kinase and a heterotrimeric protein complex consisting α, β, and γ subunits that play a key role in cellular energy homeostasis as well as one of a major regulator of post-mortem glycolysis in porcine muscle [31, 32]. Rapid alteration of AMPKα2 protein expression has been reported that was almost disappeared within 24 h post-mortem in mouse muscle [33]. We demonstrated that the expressions of GS, GSK-3β, and AMPKα were rapidly degraded within 24–36 h post-mortem and disappeared completely 48 h post-mortem (Fig 1). Caspase-3 of psoas muscle was also expeditiously decreasing within 24 h post-mortem; however, the weak intensity kept up to 60 h post-mortem. Intact protein expression intensities of the targets were calculated based on the relative control expression levels, and the decay ratio plotted against the time after post-mortem was compared to the control. The plots showed the good linear regressions of R^2^ = 0.968 and 0.991 for GS and GSK-3β in rat kidney. The R^2^ value of GS in psoas muscle also showed higher than 0.98 (Fig 1). These proteins had lesser than 24 h PMI_50_ value, which can be defined as adequate short-term PMI markers. GSK-3 is a well-characterized enzyme in post-mortem brain disease [34]. GSK-3β transcripts decreased in human brain with increasing PMI [22]. However, it is not significantly decreased with increasing PMI.

The GAPDH and PPAR-γ immunoblot expression bands degraded gradually up to 96 h post-mortem as the PMI proceeded in rat kidney or psoas muscle. The caspase-3 degradation pattern of kidney appeared rapidly; however, the PMI_50_ value was greater than 24 h post-mortem. Therefore, these proteins were defined as mid-term PMI markers (Fig 1). Transcription levels of GAPDH have been analyzed according to stability, and GAPDH has been generally used as an endogenous reference gene in rat cortex and hippocampus [35]; however, the transcript is significantly regulated in various tissues [36]. The levels of GAPDH mRNA in human brain are negative correlated with the PMI, and range from 1.5 to 45 h [22]. Caspase-3 belongs to the cysteine protease family. Caspase-3 activity and the protein levels of alpha-II spectrin and poly (ADP-ribose) polymerase, which are caspase substrates, all decrease in pig muscle during the PMI [37]. PPAR is a nuclear hormone receptor superfamily that includes PPAR-α, PPAR-β/δ, and PPAR-γ, which are involved in adipogenesis and many other biological processes [24]. Among them, PPAR-γ is the well-studied receptor. Thus, we performed further analysis with these candidate PMI-indicating proteins for the feasible application to forensic sciences.

The decaying expression patterns of GS and caspase-3 during post-mortem of rat kidney or psoas muscle were verified by *in vivo* immunohistochemical expression (Fig 2). We found no gross structural differences in kidney or psoas muscle morphologies during the process of PMI, suggesting that the overall protein degradation profiles were consistent and that the protein subsets performed in the present study were degraded during the process of PMI, at least within 96 h post-mortem. The *in vivo* degradation patterns of PMI proteins agreed with the *in vitro* biochemical analyses as increasing PMI time-points. Taken together, these results suggest that the PMI-indicating proteins including GS, GSK-3β, caspase-3, AMPKα, GAPDH, and PPAR-γ would be useful candidates to determine the PMI time-point.

Taken together, it would be interesting to consider these proteins in an extended degradation study to investigate PMI indicators and determine whether these proteins can be explained by a tissue- and/or time-specific PMI evaluation. We selected GAPDH as a mid-term PMI-indicating protein and constructed rGAPDH using *E*.*coli* expression system for rGAPDH lateral flow immunosensor (Fig 3). We designed the LFA chip for detecting the GAPDH protein in the carcass rat kidney. LFA is a simple immunoassay-based technology for point-of-care detection of target analytes (often antigenic protein) and semi-quantitation of samples [38]. Other LFAs have been designed to detect DNA or RNA in biological samples [39, 40]. However, no reports have detected PMI-indicating proteins in post-mortem samples. Only one forensic entomology study has suggested identifying a blowfly species using LFA technology instead of a DNA-sequence-based method [41]. In the present study, we developed the LFA for a possible forensic application using the same antibodies used in the sandwich ELISA. The LFA clearly detected a presence of recombinant kidney GAPDH in test line (Fig 4). In contrast, the absence of recombinant kidney GAPDH showed no reactivity in the test line, whereas the control line was clearly indicated as an internal control. This LFA was a first-time application for determining the PMI.

In conclusion, we investigated the degradation patterns of eight proteins for up to 96 h post-mortem in carcass rat kidneys and psoas muscles with five biological replicates. The proteins were divided into three groups as short-, mid-, and long-term PMI-indicating markers. GAPDH as a mid-term PMI protein of rat model system was chosen as a test PMI protein followed by performing a semi-quantitative assay using rGAPDH in LFA. We showed the good correlation of rGAPDH in a concentration-dependent manner by LFA. This would be applicable for rapid and trustworthy detection of the PMI time-point in a forensic investigation. Further sophisticated field investigation remains to be conducted for the exact time elapsed since bodies die.

## Supporting Information

S1 Fig Immunoblot images of six biological replicates of kidney PMI proteins up to 96 h post-mortem. (TIF)

S2 Fig Immunoblot images of five biological replicates of psoas muscle PMI proteins up to 96 h post-mortem. (TIF)
