# Supplementary figures and images for: Degradation of Kidney and Psoas Muscle Proteins as Indicators of Post-Mortem Interval in a Rat Model, with Use of Lateral Flow Technology
Source: PLoS One. 2016 Aug 23;11(8):e0160557. doi: 10.1371/journal.pone.0160557 (PMC4995019; doi:10.1371/journal.pone.0160557)

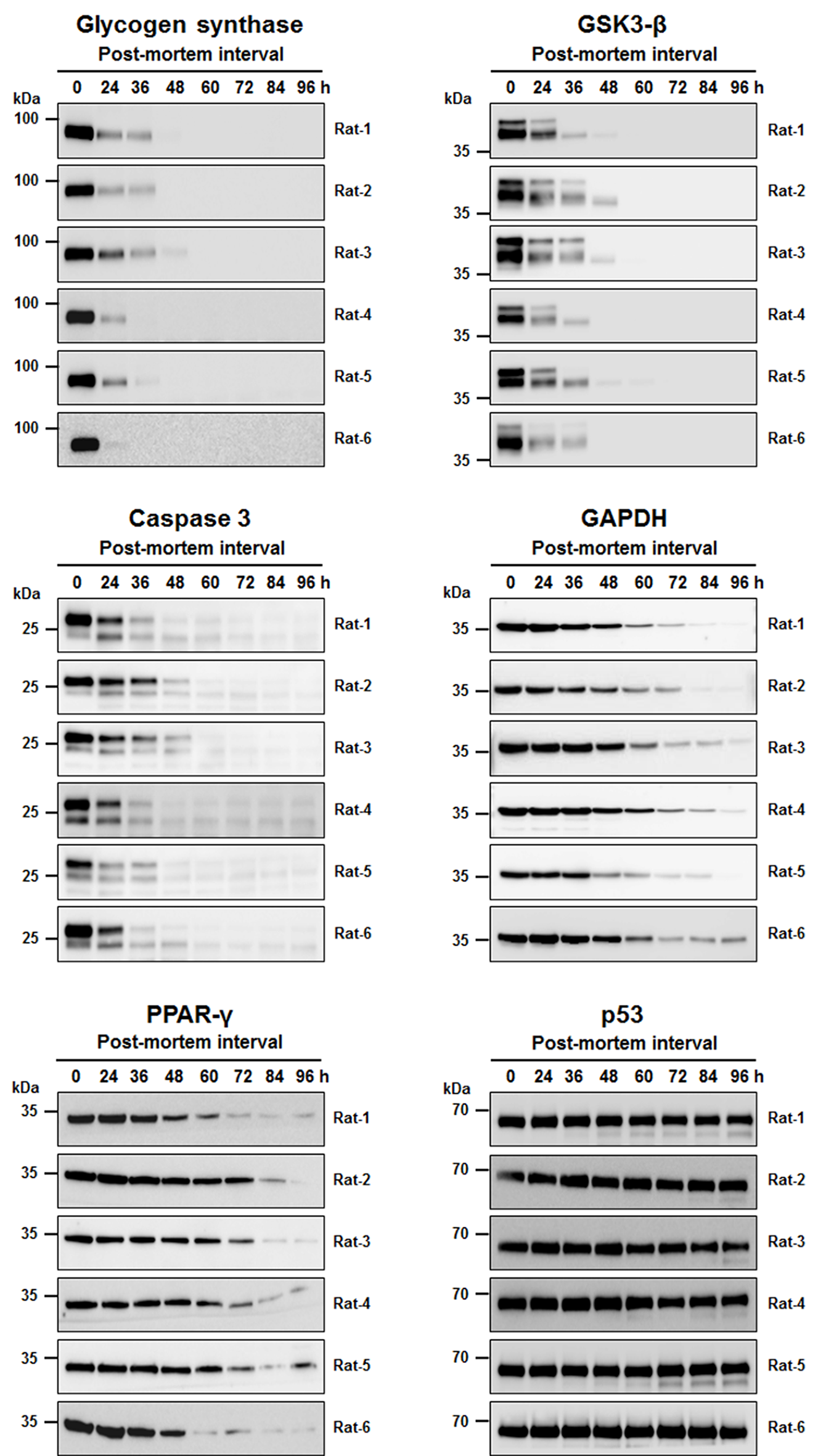

Supplement: S1 Fig — (TIF) [file pone.0160557.s001.tif]

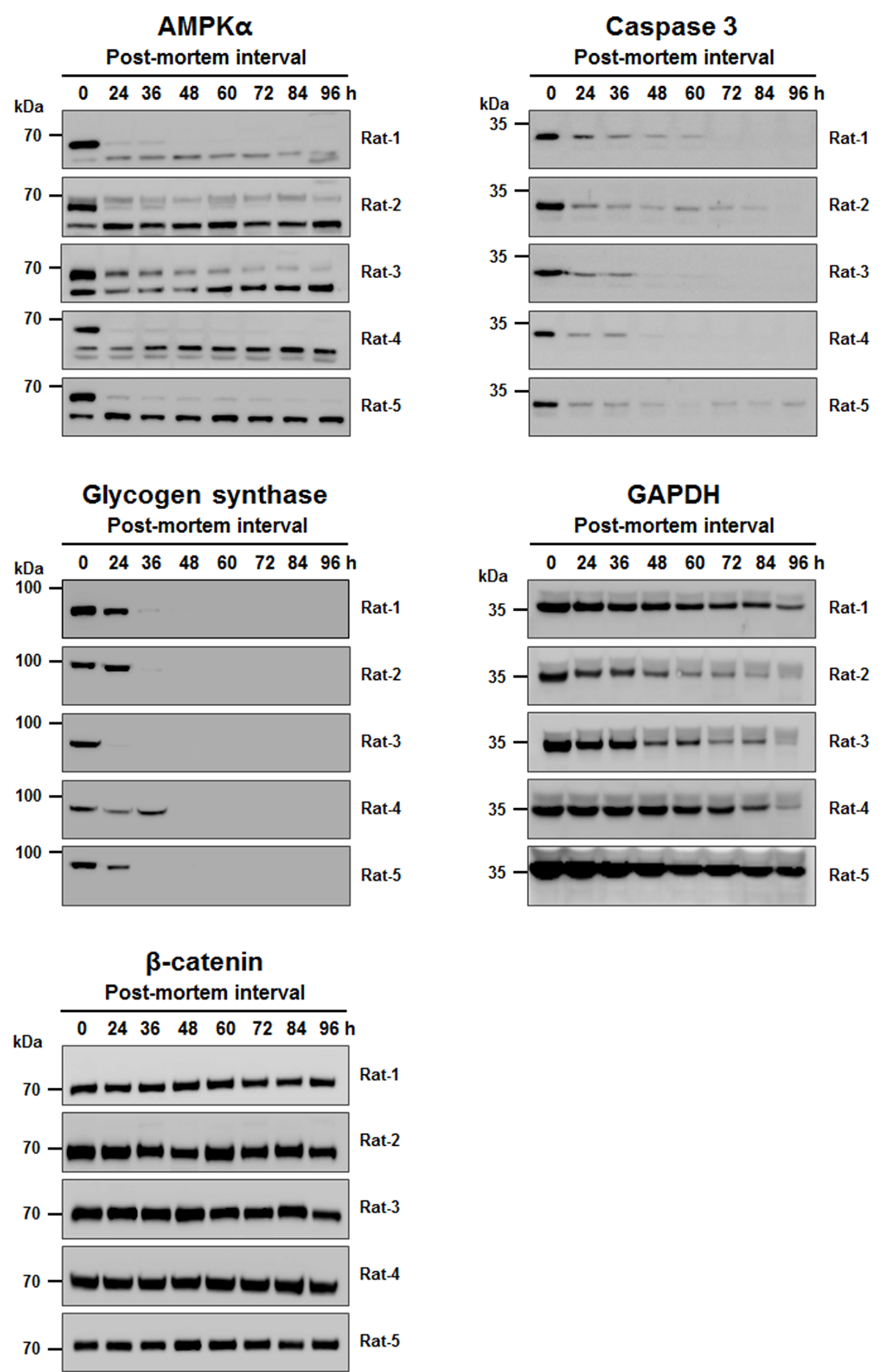

Supplement: S2 Fig — (TIF) [file pone.0160557.s002.tif]
